# Impaired tumor necrosis factor‐α secretion by CD4 T cells during respiratory syncytial virus bronchiolitis associated with recurrent wheeze

**DOI:** 10.1002/iid3.281

**Published:** 2020-01-04

**Authors:** Maleewan Kitcharoensakkul, Leonard B. Bacharier, Huiqing Yin‐Declue, Jonathan S. Boomer, Geneline Sajol, Marilyn K. Leung, Brad Wilson, Kenneth B. Schechtman, John P. Atkinson, Jonathan M. Green, Mario Castro

**Affiliations:** ^1^ The Division of Allergy, Immunology and Pulmonary Medicine, Department of Pediatrics, St. Louis Children's Hospital Washington University School of Medicine St. Louis Missouri; ^2^ The Division of Pulmonary and Critical Care Medicine, Department of Internal Medicine Washington University School of Medicine St. Louis Missouri; ^3^ The Division of Rheumatology, Department of Internal Medicine Washington University School of Medicine St. Louis Missouri; ^4^ The Division of Biostatistics Washington University School of Medicine St. Louis Missouri; ^5^ National Institutes of Health Bethesda Maryland; ^6^ The Division of Pulmonary, Critical Care and Sleep Medicine University of Kansas School of Medicine Kansas City Kansas

**Keywords:** recurrent wheeze, respiratory syncytial virus bronchiolitis, Tregs, tumor necrosis factor

## Abstract

**Background:**

Infants with severe respiratory syncytial virus (RSV) bronchiolitis have an increased risk of recurrent wheezing and asthma. We aimed to evaluate the relationships between regulatory T cell (Treg) percentage and cytokine production of in vitro‐stimulated CD4+ T cells during acute bronchiolitis and the development of recurrent wheezing in the first 3 years of life.

**Methods:**

We obtained peripheral blood from 166 infants hospitalized with their first episode of RSV‐confirmed bronchiolitis. Granzyme B (GZB) expression, and interleukin‐10, interferon‐γ, tumor necrosis factor‐α (TNF‐α), IL‐4, and IL‐5 production by in vitro anti‐CD3/CD28‐ and anti‐CD3/CD46‐activated CD4+ T cells, and percentage of peripheral Treg (CD4+CD25^hi^Foxp3^hi^) cells were measured by flow cytometry. Wheezing was assessed every 6 months. Recurrent wheezing was defined as three or more episodes following the initial RSV bronchiolitis.

**Results:**

Sixty‐seven percent (n = 111) of children had wheezing after their initial RSV infection, with 30% having recurrent wheezing. The percentage of peripheral Treg (CD4+CD25^hi^Foxp3^hi^) cells was not significantly different between the wheezing groups. Decreased TNF‐α production from anti‐CD3/CD28− and anti‐CD3/CD46− activated CD4+ T cells was observed in the recurrent wheezers, compared with nonwheezers (*p* = .048 and .03, respectively). There were no significant differences in the GZB+ CD4+ T cells and production of other inflammatory cytokines between these groups.

**Conclusions:**

We demonstrated lower TNF‐α production by in vitro stimulated CD4+ T cells during severe RSV bronchiolitis in children that subsequently developed recurrent wheezing, compared with children with no subsequent wheeze. These findings support the role of CD4+ T cell immunity in the development of subsequent wheezing in these children.

## Graphical abstract

Infants with severe respiratory syncytial virus (RSV) bronchiolitis have increased risks of recurrent wheezing and asthma. We demonstrated lower tumor necrosis factor‐α production by in vitro stimulated CD4+ T cells during severe RSV bronchiolitis in children that subsequently developed recurrent wheezing, compared with children with no subsequent wheeze.

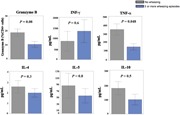

## 1 INTRODUCTION

Respiratory syncytial virus (RSV) bronchiolitis is a common cause of lower respiratory tract infection in infancy. Almost all children demonstrate seropositivity to RSV by age 2 years.^1^ Severe RSV bronchiolitis is associated with the subsequent development of recurrent wheezing, physician‐diagnosed asthma, and decreased lung function later in life.^2^, ^3^

The pathogenesis of recurrent wheezing and asthma following severe RSV bronchiolitis remains unclear. There appears to be a role of CD4+ T cells in mediating lung inflammation and pathology in RSV‐infected mice.^4^, ^5^ Immune dysregulation with an imbalance of Th1 and Th2 responses has been demonstrated in RSV‐infected mice and children.^6^, ^7^, ^8^ A recent study demonstrated that higher Th2 and Th17 cytokine levels in nasal wash samples obtained during acute RSV bronchiolitis were associated with recurrent wheezing at age one and 2 years.^9^

As Foxp3+ regulatory T cells (Tregs) play an important role in maintaining the homeostasis of the immune system,^10^, ^11^ as well as limiting the inflammatory response in the lungs during the acute phase of RSV bronchiolitis, 12, 13, 14 we hypothesized that the frequency of circulating Tregs might differ in those children subsequently developing recurrent wheezing. It has been demonstrated that infants hospitalized with severe RSV bronchiolitis have a reduction in circulating Tregs, though subsequent outcomes are unknown.^15^

In this study, the CD4+ T cells were stimulated in two conditions by using anti‐CD3/CD28 and anti‐CD3/CD46. As demonstrated in previous studies, human T cells with a Tr1‐like regulatory phenotype and Th1‐like cells can be induced in vitro by coengagement of CD3 and CD46 on CD4+ T cells, in the presence of high IL‐2, resulting in interleukin‐10 (IL‐10) secretion and expression of granzyme B (GZB).^16^, ^17^, ^18^, ^19^

The purpose of this study was to evaluate a putative relationship between circulating Foxp3+ Tregs and cytokine production of in vitro activated CD4+ T cells in infants with acute severe RSV bronchiolitis and the development of recurrent wheezing in the first 3 years of life.

## 2 METHODS

### 2.1 Participants

Peripheral blood samples were available from a subset of 179 children in a cohort of 209 children that participated in the RSV bronchiolitis in early life (RBEL)‐2 study. As previously reported,^3^ this prospective cohort of children 12 months old had their first episode of wheezing (documented by a physician) with a nasopharygeal swab positive for RSV and had bronchiolitis severe enough to require emergency department care or hospitalization. Exclusion criteria were a history of preterm birth at less than 36 weeks of estimated gestational age, previous wheezing or a diagnosis of asthma, congenital abnormalities of the heart and lung, cystic fibrosis diagnosed in the patient or immediate family, regular use of antigastroesophageal reflux medication, bronchodilators or anti‐inflammatory medications, and coinfection with other organisms. Thirty children did not have blood available for studies due to reasons that included difficult blood draws, parental refusal of blood draw attempts, or insufficient blood. Informed consent was obtained from the parents/legal guardian and approved by the Washington University Medical Center Institutional Review Board.

### 2.2 Wheezing outcomes

The parents or legal guardians of the children were contacted at 6‐month intervals by telephone to assess the occurrence of wheezing episodes in the child. We defined the occurrence of a wheezing episode each time a parent/guardian answered yes to either “Has your child had wheezing with colds?” or “Has your child had wheezing without colds?” during a follow‐up contact.^3^

### 2.3 Identification of Tregs (CD4+CD25^hi^Foxp3^hi^ cells) in peripheral blood

Tregs (CD4+CD25^hi^Foxp3^hi^ cells)were identified by the Human Regulator T‐cell Whole Blood Staining Kit. Briefly, 100 μL of fresh whole blood was incubated with fluorophore‐labeled mAbs to CD4 and CD25, followed by fixation/permeabilization. Cells were washed and incubated with anti‐human Foxp3 or IgG isotype control. Labeled cells were identified on a four‐color cytometer (BD FACSCaliber) followed by analysis on Flowjo (v9.2). To differentiate between activated T cells that also express CD25 and Foxp3 and Tregs, we analyzed the CD4+CD25^hi^Foxp3^hi^ cells by gating on CD4 expression followed by CD25^hi^ and Foxp3^hi^ expression. Tregs are presented as the percentage of CD4+ cells. See Supporting Information materials for the antibodies, media, and reagents used in the experiments.

### 2.4 Assay for CD3/CD28− and CD3/CD46‐activated CD4+ T cells

#### 2.4.1 Purification and in vitro stimulation of CD4+ lymphocytes

PBMC were isolated as previously performed^20^ and resuspended in complete Roswell Park Memorial Institute Medium (RPMI; 10% fetal calf serum [FCS], l‐glutamine, 50 U penicillin‐50 μg streptomycin/mL and at 1.5 × 10^6^ cells/mL. CD4+ T lymphocytes were isolated by magnetic bead separation using a CD4+ isolation kit according to manufacturers' protocol. Purity of CD4+ T cells was greater than 85% as determined by fluorescence‐activated cell sorting (FACS) and viability was greater than 80% as assessed by trypan blue exclusion.

Purified CD4+ T lymphocytes (5.5 × 10^4^ cells/well) were added in 55 μL of culture medium, consisting of RPMI 1640 medium plus 10% FCS and 200 mM l‐glutamine in the presence of 25 U/mL recombinant human IL‐2, placed in 384‐well plates coated with 10 μg/mL anti‐CD3 alone, 10 μg/mL anti‐CD3, and 5 μg/mL anti‐CD28, or 10 μg/mL anti‐CD3 and 10 μg/mL anti‐CD46 for generating Tr1 cells in vitro as previously described,^16^ or phosphate‐buffered saline (PBS) in medium plus rIL‐2 as a negative control. After 3 days, cell‐free supernatants were collected and frozen at −80°C for batch analysis of cytokines. CD3/CD46‐activated CD4+ T cells have a cytokine profile, IL‐10 skewed, that is distinct from that induced by CD3/CD28 stimulation but similar to Tr1 cells as previously described.^16^ After in vitro stimulation, cells were immediately analyzed by flow cytometry for GZB expression.

### 2.5 Th1/Th2 cytokine analyses

Supernatants from 3‐day cultures were thawed and batch analyzed for IL‐10, IL‐2, IL‐4, IL‐5, tumor necrosis factor‐α (TNF‐α), and interferon‐γ (IFN‐γ) using the Human Th1/Th2 cytometric bead array kit. Limits of detection of the assay are provided in the Supporting Information materials. Briefly, in 96‐well plates, 25 μL of supernatant was diluted 1:2 followed by the addition of capture beads and subsequent PE detection reagent. After 3 hours of incubation at room temperature, the beads were washed, resuspended in wash buffer and transferred to FACS tubes. Data collection was performed on FACSCalibur flow cytometry. Data analysis was performed using FCAP Array Software v3 (BD Biosciences).

### 2.6 GZB analysis

After 3 days of culture and in vitro stimulation of CD4^+^ cells with monoclonal antibodies (mAb) to CD3 and CD46 or to CD3 and CD28 or to CD3 alone, in the presence of IL‐2, cells were harvested and resuspended in PBS plus ethylenediaminetetraacetic acid solution, labeled with fluorescein isothiocyanate‐conjugated anti‐GZB Ab or isotype control then analyzed by a four‐color FACSCalibur flow cytometry using CellQuest™Pro version 5.2.1 (Becton‐Dickinson Corporation, Mountain View, CA). Results are reported as the percentage of CD4+ cells.

### 2.7 Statistical analysis

Data are reported in mean ± *SD*'s or percentages for frequencies. Results are reported in pg/mL for cytokines and mean percent of CD4+ cells for GZB expression. Cytokine level from CD4+ T cell cultures was calculated by subtracting the value of a nonstimulated well with RPMI from the well stimulated with CD3/46 or CD3/28 and then log‐transformed. Groups were compared using analysis of variance, *t* tests, and *χ* ^2^ where appropriate. A *P* value less than .05 was considered statistically significant. For the comparison on participants' characteristics, *t* test or Kruskal‐Wallis and *χ* ^2^ test were used with continuous and categorical variables, respectively. For continuous variables, we used Shapiro‐Wilk test to assess the normality of data distribution. Immunoglobulin E levels were log‐transformed. The statistical analyses were performed using SAS software, version 9.3 (SAS Institute, Cary, NC).

## 3 RESULTS

### 3.1 Demographics and wheezing episodes

Of 209 children enrolled in the RBEL‐2 cohort, 179 (85.6%) had blood drawn at entry (Figure S1). Children who did not have blood draws for the study did not differ from those with blood draws in terms of their baseline characteristics and bronchiolitis severity (Table S1). Of these 179 children, 166 (93%) subjects had adequate follow‐up data available to evaluate wheezing outcomes. Of 166 children, 160 (96%) were hospitalized and the rest required only treatment in the emergency room. The average age of entry to the study was 4.2 ± 2.9 months and the average age at follow‐up was 28.8 ± 15.6 months. Approximately half of the cohort were Caucasians and 60% were male. Majority of children in this cohort are from atopic family with 80% had first‐degree relatives with atopic diseases. During the first 3 years of life, 111 (67%) children experienced at least one wheezing episode; 35 (21%) had one wheezing episode, 26 (16%) had two wheezing episodes and 50 (30%) had recurrent wheezing defined as three or more episodes. Compared to children with no subsequent wheezing, the recurrent wheezing group had a higher proportion of males and non‐Caucasian races (Table 1). The recurrent wheezing group had a longer duration of follow‐up (31.3 ± 11.9 vs 23.1 ± 16.8 months, *P* = .007) and a shorter duration of maternal pregnancy (38.7 ± 1.2 weeks in recurrent wheezing group vs 39.0 ± 1.2 weeks in no wheezing group, *P* = .01). Due to differences in gender, race, and duration of follow‐up between the wheezing groups which potentially confound the wheezing outcomes, these variables were included as covariates in the analysis of the primary outcome of the study. The overall prevalence of physician‐diagnosed asthma was 15%, and the recurrent wheezing group had a significantly higher prevalence of MD‐diagnosed asthma compared to the no wheezing group (34% vs 2%, *P* < .0001). The average duration between the onset of bronchiolitis symptoms and blood collection was 3.5 ± 1.9 days. There were no differences on the timing of blood collection among wheezing groups (Tables 1 and S2).

**Table 1:** Characteristics and wheezing outcome of children following severe RSV bronchiolitis during infancy

Characteristics	All (n = 166)	No wheezing (n = 55)	≥3 wheezing episodes (n = 50)	*P* value (No vs ≥3 wheezing)
Demographics				
Age at RSV (study entry), m	4.2 ± 2.9	3.9 ± 3.1	4.3 ± 2.8	0.2
Age at follow‐up, m	28.8 ± 15.6	26.4 ± 18.0	34.8 ± 12.0	0.01
Duration of follow‐up, m	24.7 ± 15.1	23.1 ± 16.8	31.1 ± 11.9	0.01
Male sex, %	98 (59.0)	27 (49.0)	35 (70.0)	0.03
Caucasians, %	77 (46.3)	32 (58.1)	19 (38.0)	0.04
Pregnancy history				
Duration of pregnancy, w	38.7 ± 1.3	39.0 ± 1.2	38.4 ± 1.2	0.01
Birth weight, g	3300 ± 473	3379 ± 420	3257 ± 518	0.2
Birth length, cm	50.4 ± 2.9	50.2 ± 3.1	50.7 ± 2.9	0.6
Hospitalization data				
Length of stay, d	3.8 ± 2.0	3.7 ± 1.8	4.0 ± 2.5	1.0
Lowest SaO_2_, %	90.7 ± 5.4	91.0 ± 4.4	90.4 ± 7.2	0.4
Bronchiolitis severity score	7.5 ± 2.2	7.4 ± 2.1	7.3 ± 2.2	0.9
Durationbetween onset of symptoms and blood draw, days	3.5 ± 2.9	3.2 ± 2.7	3.1 ± 2.6	0.9
Family history				
History of first‐degree relatives with asthma, %	84 (50.9)	22 (40.0)	27 (55.1)	0.1
History of first‐degree relatives with atopic diseases, %	135 (81.8)	42 (76.3)	40 (81.6)	0.5
Other histories and exposures				
Personal history of eczema	22 (13.4)	6 (10.9)	10 (20.4)	0.2
Intraute rine exposure to cigarette smokes	41 (24.8)	17 (31.4)	10 (20.0)	0.2
Postnatal exposure to cigarette smoke	82 (49.4)	25 (45.4)	26 (52.0)	0.5
History of daycare attendance	50 (30.1)	17 (30.9)	13 (26.0)	0.6
Laboratory tests at baseline				
Baseline IgE (IU/mL) (n = 95)	32 ± 116	36 ± 107	22 ± 60	0.4
Baseline eosinophils (%) (n = 87)	1.5 ± 1.8	1.6 ± 2.6	1.3 ± 1.2	0.7

*Note*: Data are expressed as means ± SDs, except as noted.

Abbreviations: IgE, immunoglobulin E; RSV, respiratory syncytial virus.

### 3.2 Percent of Tregs (CD4+CD25^hi^Foxp3^hi^) cells in blood during acute illness in the recurrent wheezing group

The average percentage of CD4+CD25^hi^Foxp3^hi^ cells in peripheral blood was 0.99 ± 0.51% CD4+ cells for recurrent wheezers and 1.07 ± 0.54% CD4+ cells for never wheezers (Table 2). Even after adjustment for covariates of age at follow‐up, gender, and race, there was no statistical difference in children with recurrent wheezing compared to never wheezers. As activated CD4+ T cells also express CD25, the IL‐2R, and Foxp3, the overall percentage of CD4+CD25+Foxp3+ T cells in recurrent wheezers vs never wheezers were assessed. Never wheezers had slightly increased CD4+CD25+Foxp3+ T cells (5.9 ± 2.3% CD4+ cells) compared to wheezers (5.0 ± 1.9% CD4+ cells, *P* = .051) which became significant after adjustment for covariates; *P* = .03.

**Table 2:** Peripheral blood CD4+CD25^hi^Foxp3^hi^ cells and granzyme B expression and cytokine production from CD3/CD46‐activated and CD3/CD28‐activated CD4+ cells during severe RSV bronchiolitis in infancy by wheezing outcome

	No wheezing	≥3 wheezing episodes	Unadjusted *P* value^a^	Adjusted *P* value^b^
CD4+CD25^hi^Foxp3^hi^ cells (N = 101, no wheezing n = 52, ≥3 wheezing n = 49)				
% CD4^+^ cells	1.07 ± 0.54	0.99 ± 0.51	0.6	0.5
CD3/CD46‐activated CD4+ T cells (N = 104, no wheezing n = 50, ≥3 wheezing n = 44)				
Granzyme B (% CD4+)	18.7 ± 17.6	10.4 ± 13.6	0.009	0.08
IL‐10, pg/mL	178 ± 323	100 ± 245	0.2	0.5
TNF‐α, pg/mL	354 ± 454	155 ± 237	0.01	0.048
IFN‐γ, pg/mL	881 ± 3,326	1,362 ± 3,605	0.5	0.6
IL‐5, pg/mL	86.6 ± 270	55.0 ± 148	1.0	0.8
IL‐4, pg/ml	2.6 ± 4.2	2.0 ± 2.7	0.8	0.3
CD3/CD28‐activated CD4+ T cells (N = 104, no wheezing n = 50, ≥3 wheezing n = 44)				
Granzyme B (% CD4+)	6.8 ± 8.0	5.3 ± 8.0	0.3	0.4
IL‐10, pg/mL	64.4 ± 114	51.5 ± 109	0.2	0.3
TNF‐α, pg/mL	239 ± 328	142 ± 280	0.009	0.03
IFN‐γ, pg/mL	1,263 ± 1,488	1,488 ± 3,397	0.5	0.5
IL‐5, pg/mL	106 ± 313	80.6 ± 141	0.7	0.9
IL‐4, pg/mL	2.8 ± 3.2	3.5 ± 3.5	0.4	0.9

*Note*: Data were subtracted from negative control conditions (PBS) and negative data were adjusted to zero, then log‐transformed for statistical analysis.

Abbreviations: IFN‐γ, interferon‐γ; IL, Interleukin; TNF‐α, tumor necrosis factor‐α.

^a^ Comparison between no wheezing and ≥3 wheezing episodes.

^b^ Adjusting variables including age at follow‐up, race, and gender.

### 3.3 Decreased TNF‐α production by in vitro CD3/CD28‐activated CD4+ T cells and CD3/CD46‐activated CD4+ T‐cells in the recurrent wheezing group

**Figure 1:**
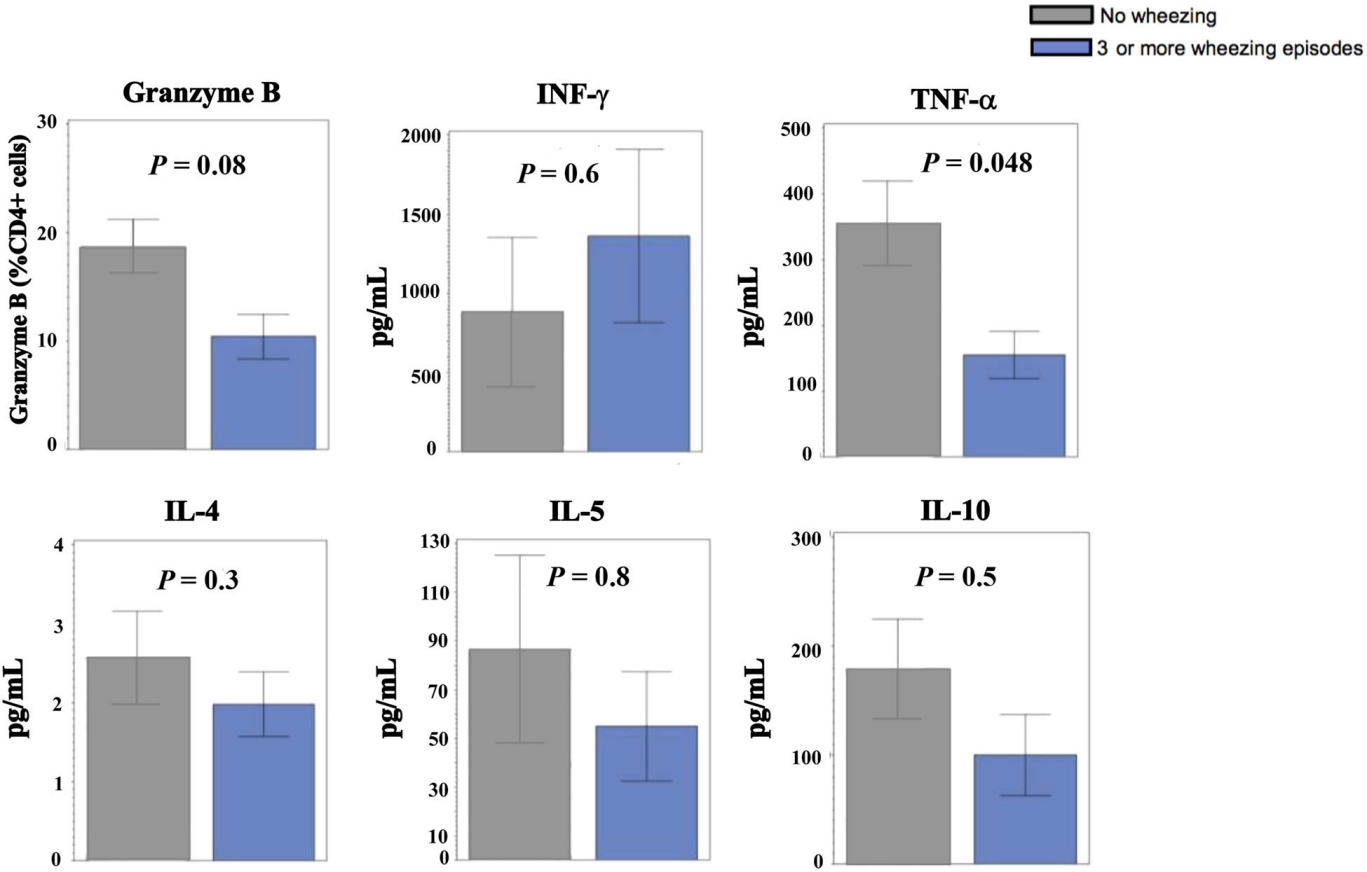
Granzyme B expression and cytokine productions by CD3/CD46‐activated CD4+ T cells in vitro in no wheezing and recurrent wheezing groups. *Bar graphs with mean ± one standard error are shown. Data were subtracted from negative control conditions, and log‐transformed for statistical analysis. IFN‐γ, interferon‐γ; IL, Interleukin; TNF‐α, tumor necrosis factor‐α

The percentage of CD3/CD46‐activated CD4+ T cells, expressed as % of CD4+ T cells that are positive for GZB was lower in the recurrent wheezing group compared to never wheezers (10.4 ± 13.6 vs 18.7 ± 17.6% CD4+ cells, *P =* .009; Figure 1 and Table 2). However, after adjustment for covariates, this was not statistically different (*P* = .08). CD3/CD46‐activated CD4+ T cells from children in the recurrent wheezing group produced lower levels of TNF‐α than never wheezers (155 ± 237 vs 354 ± 454 pg/mL; *P* = .01 unadjusted, *P* = .048 adjusted). Diminished TNF‐α production was observed between any wheezers relative to never wheezers (*P* = .01, Table S3). IL‐10 and IFN‐γ production did not significantly differ between the recurrent wheezing group and the non‐wheezing group. The production of IL‐10, IL‐4, and IL‐5 from CD3/CD46‐activated CD4+ T cells did not significantly differ by wheezing category.

CD3/CD28‐activated CD4+ T cells also produced significantly less TNF‐α in the recurrent wheezing group compared to never wheezers (142 ± 280 vs 240 ± 328 pg/mL; *P* = .009 unadjusted, *P* = .03 adjusted), Table 2. Similar trends were observed between never wheezers and any wheezers (240 ± 328 vs 146 ± 254 pg/mL, *P* = .02). There were no differences between the groups in production of cytokines, including IFN‐γ, IL‐10, IL‐4, and IL‐5.

## 4 DISCUSSION

Although it is known that infants with severe RSV bronchiolitis have increased risks of recurrent wheezing and asthma in later life, previous literature on roles of innate and adaptive cytokines in the pathogenesis of subsequent wheeze following acute severe RSV bronchiolitis have been limited.^9^ In our study, we have demonstrated impaired TNF‐α production from in vitro CD3/CD28‐activated CD4+ T cells and CD3/CD46‐activated CD4+ T cells during acute illness in children who developed recurrent wheezing following severe RSV bronchiolitis. Previous studies in humans were performed to describe the cytokine profiles during RSV bronchiolitis compared to normal controls or other types of respiratory viruses, or to evaluate the association of cytokine profiles and the severity of RSV bronchiolitis in young children; and the findings from prior studies suggest a role for CD4+ T cells in the airway inflammation in these children.^8^, ^20^, ^21^, ^22^, ^23^, ^24^, ^25^, ^26^ However, the results on the Th1/Th2 imbalance is conflicting with no mechanistic data for the production of cytokines after in vitro stimulation of CD4+ T cells followed by correlation to wheezing outcomes presented. We previously showed that severe RSV infection in early life was associated with a high incidence of asthma and eczema; however, it was not associated with a Th2 phenotype in the peripheral blood.^27^ A recent birth cohort study by Turi, et al have demonstrated infants who are at increased risks for recurrent wheeze in the first and second year following acute RSV infection had a unique pattern of nasal immune response characterized by lower non‐IFN antiviral immune‐response mediators including TNF‐α and higher type‐2 and type‐17 cytokines.^9^

TNF‐α is a proinflammatory cytokine that plays roles in neutrophil and eosinophil recruitment to the airway.^28^ Higher TNF‐α levels in airway secretions have been observed in mice and human with RSV infection.^7^, ^29^ However, it has been shown that lower blood TNF‐α levels in infants with RSV bronchiolitis were associated with greater disease severity and a longer hospital stay.^24^, ^30^ Our finding is consistent with the study by Turi et al^9^ evaluating nasal immune response phenotypes in infants with RSV infection in which they demonstrated low non‐IFN antiviral immune response, including TNF‐α, as significantly associated with recurrent wheezing in the first and second‐year following RSV infection. In the upper airways, a decrease in IL‐10 potentiates an increase in the TNF‐α response which controls the viral infection.^31^ As diminished TNF‐α observed in recurrent wheezers after both stimulation conditions from peripheral blood‐derived CD4+ T cells and the significant differences in the levels of TNF‐α among four wheezing categories, evidence suggests that (a) CD4+ T cells may be impaired in their production of TNF‐α during RSV infection and (b) TNF‐α may play roles in the pathogenesis of airway inflammation and subsequent wheezing following severe RSV bronchiolitis in children.

We did not discern a difference in IFN‐γ levels between the wheezing groups after in vitro stimulation which is consistent with our previous study.^27^ The data on the role of IFN‐γ in RSV infection are conflicting. The IFN‐γ response in children less than 6 months old have a reduced IFN‐γ response which contributes to the increased incidence of RSV in the younger age group.^31^ One study has shown that IFN‐γ in nasal secretions might be protective of disease severity in RSV bronchiolitis^32^, ^33^; however, the opposite was seen in a larger cohort.^34^

The Th2 response in RSV is required for antibody production and to limit Th1 induced inflammation.^5^ Although studies in mice have demonstrated an important role for IL‐10 in limiting lung inflammation in RSV bronchiolitis,^35^, ^36^ the current evidence on the protective role of IL‐10 in RSV‐infected humans is conflicting.^5^, ^37^, ^38^ The production of IL‐10, IL‐4, and IL‐5 from in vitro stimulated T‐cells in our study was not statistically different between nonwheezers and wheezers, consistent with prior studies. Further understanding mechanisms of the immune response to RSV infection that lead to the development of recurrent wheezing and asthma in these children especially in the lung and airways where the virus resides is needed.

Granzymes are proteinases that are expressed by various immune cells including T cells.^39^, ^40^ Children with severe RSV bronchiolitis have increased the production of granzymes from immune cells,^41^ and Tregs help control lung inflammation during RSV infection in a GZB‐dependent manner.^42^ However, after adjusting with covariates, we did not find a significant difference in the percentage of GZB+CD4+T cells after in vitro stimulation of CD4+ T cells between the recurrent wheezing group and those who never wheezed.

The percentage of peripheral Tregs (CD4+CD25^hi^Foxp3^hi^) cells in children in our cohort is comparable to a previous report.^43^ The percentage of Tregs in adult humans represent 4% to 10% of CD4+ T cells, while this percentage decreases in children, suggesting that small changes can have dramatic impacts on immune function.^44^ Previous studies have demonstrated that host T cell responses play a role in causing lung pathology following RSV bronchiolitis.^12^, ^35^ We did find a small reduction in the number of CD4+CD25+Foxp3+ activated cells in children with recurrent wheezing, compared to children who never wheezed. The slight reduction in both Treg (CD4+CD25^hi^Foxp3^hi^) and activated CD4+CD25+Foxp3+ T cells could be due to a recruitment of these cells to airway, apoptosis or their plasticity in these children with acute severe RSV bronchiolitis. Although this could reflect the severity of infection, we did not find significant differences in bronchiolitis severity (as measured by bronchiolitis severity scores, lowest saturation during hospitalization and duration of hospital stay) and wheezing outcome in this cohort, Table 1. Although there was a statistical difference between the percentage of activated T cells (CD4+CD25+Foxp3+) cells between the recurrent wheezing group and non‐wheezing group, the percentage change is small. The lower percentage of activated T cells (CD4+CD25+Foxp3+) in the recurrent wheezers compared to those whom never wheezed is consistent with the lower TNF‐α secretion upon in vitro stimulation in these children. However, functional studies of these T‐cells should be performed in a future cohort as the suppressive function and cytokine profile could be impaired despite the similar percentage of CD4+ T cells.

Our cohort has several unique characteristics. It is one of the largest prospective cohorts in children with severe RSV bronchiolitis in infancy with comprehensive characterization at baseline, regular follow‐up visits, even distribution among Caucasians and African‐Americans, and a high retention rate of 92%. In this cohort, the majority of children with acute severe RSV bronchiolitis in infancy developed subsequent wheezing and approximately one‐third experienced recurrent wheezing. Our findings are consistent with previous studies indicating that wheezing is a common outcome in children with a history of severe RSV bronchiolitis.^45^, ^46^ Due to a lack of objective measurement for asthma in young children, and because recurrent wheezing is a finding closely associated with the diagnosis of asthma, it was used as the primary outcome in our study.

Limitations of the present study include the fact that experiments for CD4+ T cells were performed in vitro on peripheral blood‐derived cells and this might not reflect biological phenomena occurring in vivo, especially at the site of RSV infection like the lung and upper airways. Although T cells from the airways might better reflect lung pathology, we could not obtain lung specimens from these infants without risks. We also did not obtain specimens from age‐matched normal controls for comparison, as RBEL is a prospective observational cohort of children exposed to severe RSV allowing us to use children who did not develop recurrent wheezing as a comparison group. We also did not assess baseline CD4+ cell phenotype and function before RSV bronchiolitis as this was not a birth cohort. As the recurrent wheezers had a significant longer period of follow‐up, it is possible that the never wheezers could develop wheezing later in life. The wheezing outcomes were assessed by telephone interview, so this may not be an accurate parameter of the outcome. However, wheezing is a common outcome used in a number of studies as the majority of these wheezing episodes may not require medical attention.^9^, ^47^ Due to the limitation of blood that we can draw from infants, we did not measure the plasma cytokines in our subjects to determine the correlation with the in vitro CD4+ functional measures and did not have an adequate data to provide additional insights into the kinetics of these cytokines. The limited volume of blood in infants also prevented absolute cell counts from being determined. Additional T cell populations and cytokines could play an important role in the T cell‐dependent response during airway inflammation and exacerbation of bronchial asthma in this population.^48^, ^49^ Lastly, as the cytokines measured in the study are certainly correlated, the significant finding could be a result of multiple comparisons, so the studies should be repeated in a different, preferably larger cohort.

In summary, we have demonstrated lower TNF‐α production by in vitro stimulated CD4+ T cells during severe RSV bronchiolitis in children who subsequently developed recurrent wheezing in the first 3 years of life. This supports the role of CD4+ T‐cell immunity in the development of recurrent wheezing in these children.

## CONFLICT OF INTERESTS

The authors declare that there are no conflict of interests.

## ETHICAL STATEMENT

The study was performed according to the Declaration of Helsinki and approved by the Washington University Medical Center Institutional Review Board. The written informed consent was obtained from the participants' parents/legal guardians.

## Supporting information

Supplementary information

Supplementary information

Supplementary information

Supplementary information

## Data Availability

The data that support the findings of this study are available from the corresponding author upon reasonable request.
