# Supplementary material for: Impaired tumor necrosis factor‐α secretion by CD4 T cells during respiratory syncytial virus bronchiolitis associated with recurrent wheeze
Source: Immun Inflamm Dis. 2020 Jan 4;8(1):30–9. doi: 10.1002/iid3.281 (PMC7016853; doi:10.1002/iid3.281)
Supplement: Supplementary file 1 — Supplementary information [file IID3-8-30-s001.docx]

**Supplement tables**

**Suppl. Table 1** Baseline characteristics of children with severe RSV bronchiolitis in RBEL-II that did or did not have blood draw for the study

| **Characteristics** | **Children in the cohort with blood draw**  **(n=179)** | **Children in the cohort without blood draw (n=30)** | ***P* value** |
| --- | --- | --- | --- |
| **Demographics** | | | |
| Age at RSV (study entry, [m]) | 4.2 ± 2.9 | 4.4 ± 3.2 | 0.7^1^ |
| Male sex (%) | 98 (59.1) | 23 (53.5) | 0.5^2^ |
| Caucasians (%) | 77 (46.4) | 18 (41.9) | 0.6^2^ |
| **Pregnancy history** | | | |
| Duration of pregnancy, [w] | 38.7 ± 1.3 | 38.8 ± 1.5 | 0.6^1^ |
| Birth weight, [g] | 3300.1 ± 473.9 | 3241.7 ± 529.3 | 0.5^1^ |
| Birth length, [cm] | 50.4 ± 2.9 | 50.6 ± 3.3 | 0.7^1^ |
| **Hospitalization data** | | | |
| Length of stay [d] | 3.8 ± 2.0 | 4.1 ± 4.5 | 0.5^1^ |
| Lowest SaO_2_ (%) | 90.7 ± 5.4 | 87.9 ± 15.9 | 0.3^4^ |
| Bronchiolitis severity score | 7.5 ± 2.2 | 7.5 ± 2.5 | 0.9^1^ |
| **Family history** | | | |
| History of first-degree relatives with asthma (%) | 84 (50.9) | 22 (51.1) | 1.0^2^ |
| History of first-degree relatives with atopic diseases (%) | 135 (81.8) | 30 (69.8) | 0.08^2^ |
| Personal history of eczema | 22 (13.4) | 13 (16.9) | 0.5^2^ |
| Intrauterine exposure to cigarette smokes | 41 (24.9) | 9 (20.9) | 0.6^2^ |
| Postnatal exposure to cigarette smoke | 82 (49.4) | 25 (58.1) | 0.3^2^ |
| History of daycare attendance | 50 (30.1) | 7 (16.3) | 0.07^2^ |
| **Laboratory studies** | | | |
| Baseline IgE (IU/ml) (n=110) | 32.4 ± 116.3 | 21.3 ± 29.9 | 0.9^3^ |
| Baseline eosinophils  ( %) (n=102) | 1.5 ± 1.8 | 2.5 ± 3.8 | 0.3^4^ |

1. t-test
2. Chi-square
3. t-test performed on log-transformed data
4. t-test with Satterthwaite correction

**Suppl Table 2 :** Characteristics and wheezing outcome of children following severe RSV bronchiolitis during infancy

| **Characteristics** | **All**  **(n=166)** | **No wheezing**  **(n=55)** | **Only 1 wheezing episode**  **(n=35)** | **Only 2 wheezing episodes**  **(n=26)** | **≥3 wheezing episodes**  **(n=50)** | **Overall *P*-value** |
| --- | --- | --- | --- | --- | --- | --- |
| **Demographics** | | | | | | |
| Age at RSV (study entry, [m]) | 4.2 ± 2.9 | 3.9 ± 3.1 | 4.1 ± 2.7 | 5.0 ± 2.8 | 4.3 ± 2.8 | 0.4^1^ |
| Age at follow-up, [m]* | 28.9 ± 15.3 | 27.0 ± 17.5 | 26.9 ± 14.6 | 23.5 ± 13.0 | 35.4 ± 12.2 | 0.003^1^ |
| Duration of follow-up, [m])* | 24.7 ± 15.1 | 23.1 ± 16.8 | 22.8 ± 14.8 | 18.5 ± 13.4 | 31.1 ± 11.9 | 0.002^1^ |
| Male sex (%) | 98 (59.0) | 27 (49.0) | 19 (54.2) | 17 (65.3) | 35 (70.0) | 0.1^2^ |
| White race (%) | 77 (46.3) | 32 (58.1) | 16 (45.7) | 10 (38.4) | 19 (38.0) | 0.2^2^ |
| **Pregnancy history** | | | | | | |
| Duration of pregnancy, [w] | 38.7 ± 1.3 | 39.0 ± 1.2 | 38.9 ± 1.4 | 38.5 ± 1.6 | 38.4 ± 1.2 | 0.1^1^ |
| Birth weight, [g] | 3300 ± 474 | 3379 ± 420 | 3276 ± 523 | 3248 ± 422 | 3258 ± 518 | 0.5^1^ |
| Birth length, [cm] | 50.4 ± 2.9 | 50.2 ± 3.1 | 49.9 ± 3.0 | 50.8 ± 2.8 | 50.7 ± 2.9 | 0.6^1^ |
| **Hospitalization data** | | | | | | |
| Length of stay,[d] | 3.8 ± 2.0 | 3.7 ± 1.8 | 3.6 ± 1.3 | 3.7 ± 2.0 | 4.0 ± 2.5 | 0.8^1^ |
| Lowest SaO_2_ (%) | 90.7 ± 5.4 | 91.0 ± 4.4 | 89.9 ± 4.2 | 91.4 ± 4.7 | 90.4 ± 7.2 | 0.7^1^ |
| Bronchiolitis severity score | 7.5 ± 2.2 | 7.4 ± 2.1 | 8.3 ± 2.0 | 7.3 ± 2.3 | 7.3 ± 2.2 | 0.2^1^ |
| Duration between onset of symptoms and blood draw | 3.5 ± 2.9 | 3.2 ± 2.7 | 4.1 ± 3.7 | 3.9 ± 2.8 | 3.1 ± 2.6 | 0.4^1^ |
| **Family history** | | | | | | |
| History of first-degree relatives with asthma | 84 (50.9) | 22 (40.0) | 19 (54.3) | 16 (61.5) | 27 (55.1) | 0.2^2^ |
| History of first-degree relatives with atopic diseases | 135 (81.8) | 42 (76.4) | 32 (91.4) | 21 (80.8) | 40 (81.6) | 0.4^2^ |
| Personal history of eczema | 22 (13.4) | 6 (10.9) | 5 (14.7) | 1 (3.9) | 10 (20.4) | 0.2^2^ |
| Intrauterine exposure to cigarette smokes | 41 (24.9) | 17 (31.5) | 9 (25.7) | 5 (19.2) | 10 (20.0) | 0.5^2^ |
| Postnatal exposure to cigarette smokes | 82 (49.4) | 25 (45.5) | 17 (48.6) | 14 (53.9) | 26 (52.0) | 0.9^2^ |
| History of daycare attendance | 50 (30.1) | 17 (30.9) | 11 (31.4) | 9 (34.6) | 13 (26.0) | 0.9^2^ |
| **Laboratory tests at baseline** | | | | | | |
| Baseline IgE (IU/ml) (n=95) | 32.4 ± 116.3 | 35.6 ± 107.5 | 10.5 ± 16.5 | 90.8 ± 263.8 | 21.8 ± 59.7 | 0.8 ^1^ |
| Baseline eosinophils (%) (n=87) | 1.5 ± 1.8 | 1.6 ± 2.6 | 1.5 ± 1.4 | 1.8 ± 0.9 | 1.3 ± 1.2 | 0.9^1^ |

1 Analysis of variance

2 Chi-square

* *P*-value < 0.05

**Suppl. Table 3**: Peripheral blood Foxp3+ CD25+ CD4+ cells and granzyme B expression and cytokine production from CD3/CD46-activated CD4^+^ cells and CD3/CD28-activated CD4^+^ cells during severe RSV bronchiolitis in infancy by wheezing outcome. Data shown in mean ± standard deviation

| **Variables** | **No wheezing** | **≥ 1wheezing episodes** | **1 wheezing episode** | **2 wheezing episodes** | **≥3 wheezing episodes** | **P-value^1^** |
| --- | --- | --- | --- | --- | --- | --- |
| **Foxp3+ CD25+ CD4+ cells (No wheezing n=52, 1 wheezing episode** **n=35, 2 wheezing episodes n=26, ≥ 3 wheezing episodes n=49)** | | | | | | |
| % CD4+ cells | 5.9 ± 2.3 | 5.1 ± 2.0 | 5.0 ± 2.0 | 5.4 ± 2.0 | 5.0 ± 1.9 | 0.04 |
| **CD3/46-activated CD4+ T cells (No wheezing n=50, 1 wheezing episode n=34, 2 wheezing episodes n=24, ≥ 3 wheezing episodes n=44)** | | | | | | |
| Granzyme B  (% CD4+) | 18.7 ± 17.6 | 12 ± 13.1 | 11.9 ± 12.4 | 15.0 ± 13.3 | 10.4 ± 13.6 | 0.03 |
| IL-10 (pg/ml) | 179 ± 323 | 72.3 ± 177 | 48.8 ± 79.9 | 54.2 ± 113 | 100 ± 246 | 0.1 |
| TNF-α (pg/ml) | 355 ± 454 | 177 ± 326 | 147 ± 254 | 258 ± 510 | 155 ± 237 | 0.01 |
| IFN-γ (pg/ml) | 881 ± 3326 | 881 ± 332 | 325 ± 1165 | 634 ± 2396 | 1362 ± 3605 | 0.2 |
| IL-5 (pg/ml) | 86.6 ± 271 | 32.8 ± 100 | 13.9 ± 24.3 | 19.0 ± 23.6 | 55.0 ± 148 | 0.5 |
| IL-4 (pg/ml) | 2.6 ± 4.2 | 1.7 ± 2.6 | 1.3 ± 2.6 | 1.8 ± 2.5 | 2.0 ± 2.7 | 1.0 |
| **CD3/28-activated CD4+ T cells (No wheezing n=50, 1 wheezing episode n = 34, 2 wheezing episodes n=24, ≥ 3 wheezing episodes n=44)** | | | | | | |
| Granzyme B  (% CD4+) | 6.8 ± 8.0 | 5.6 ± 9.5 | 5.5 ± 6.7 | 6.4 ± 14.4 | 5.3 ± 8.0 | 0.2 |
| IL-10 (pg/ml) | 64.4 ± 115 | 45.0 ± 115 | 50.5 ± 89.5 | 25.6 ± 113 | 51.5 ± 109 | 0.1 |
| TNF-α (pg/ml) | 240 ± 328 | 146 ± 254 | 160 ± 176 | 135 ± 302 | 142 ± 281 | 0.02 |
| IFN-γ (pg/ml) | 1264 ± 3255 | 995 ± 2707 | 610 ± 2119 | 637 ± 1828 | 1488 ± 3397 | 0.3 |
| IL-5 (pg/ml) | 107 ± 314 | 61.9 ± 111 | 55.0 ± 96.5 | 37.4 ± 43.6 | 80.6 ± 141 | 1.0 |
| IL-4 (pg/ml) | 2.8 ± 3.2 | 3.3 ± 3.9 | 3.2 ± 4.7 | 2.9 ± 3.5 | 3.5 ± 3.5 | 0.6 |

1 P-value for comparison between no wheezing and ≥ 1 wheezing episode

^+^ Analysis of variance

^++^ Untransformed data in table. Data was log transformed for analysis of variance.
