# Supplementary material for: Impaired tumor necrosis factor‐α secretion by CD4 T cells during respiratory syncytial virus bronchiolitis associated with recurrent wheeze
Source: Immun Inflamm Dis. 2020 Jan 4;8(1):30–9. doi: 10.1002/iid3.281 (PMC7016853; doi:10.1002/iid3.281)
Supplement: Supplementary file 2 — Supplementary information [file IID3-8-30-s002.docx]

**Supplemental text file**

***Antibodies, media, and reagents***

Identification of Foxp3+ Tregs in peripheral blood

- Human Regulatory T-cell Whole Blood staining Kit (e Bioscience)
- BD FACSCaliber (BD Biosciences)

Purification and in vitro stimulation of CD4+ lymphocytes

- CD4+ Isolation kit (Miltenyl Biotec, Auburn, CA)
- RPMI 1640 medium (Sigma-Aldrich, St Louis, MO)
- recombinant human IL-2 (Chiron, Emeryville, CA)
- 384-well plates (Corning, NY)
- Monoclonal antibodies (mAbs) reactive with human CD3 (OKT3, ATCC, Manassas, VA), CD46 (TRA-2-10), and CD28 (CD28.2, BD Biosciences, San Jose, CA)

Th1/Th2 cytokines

- Human Th1/Th2 cytometric bead array kit (CBA, BD Biosciences)
- 96-well plates (Corning, NY)

Fluorophore-conjugated mAbs were used from eBioscience (San Diego, CA): allophycocyanine (APC)-conjugated anti-CD25 (clone BC96), fluorescein isothiocyanate (FITC)-conjugated anti-CD4 (clone RPA-T4), phycoerythrin (PE)-conjugated anti-Foxp3 (clone PCH101), mouse isotype-matched control mAb and from BD Pharmingen (San Jose, CA): FITC-conjugated anti-GZB (clone GB11) and FITC anti-human IgG1.

***Th1/Th2 cytokine analyses***

The limit of detection was 4.5, 2.6, 4.9, 2.4, 3.8, and 3.7 pg/ml for IL-10, IL-2, IL-4, IL-5, TNF-α, and IFN-γ, respectively.
