# Supplementary figures and images for: Impaired tumor necrosis factor‐α secretion by CD4 T cells during respiratory syncytial virus bronchiolitis associated with recurrent wheeze
Source: Immun Inflamm Dis. 2020 Jan 4;8(1):30–9. doi: 10.1002/iid3.281 (PMC7016853; doi:10.1002/iid3.281)

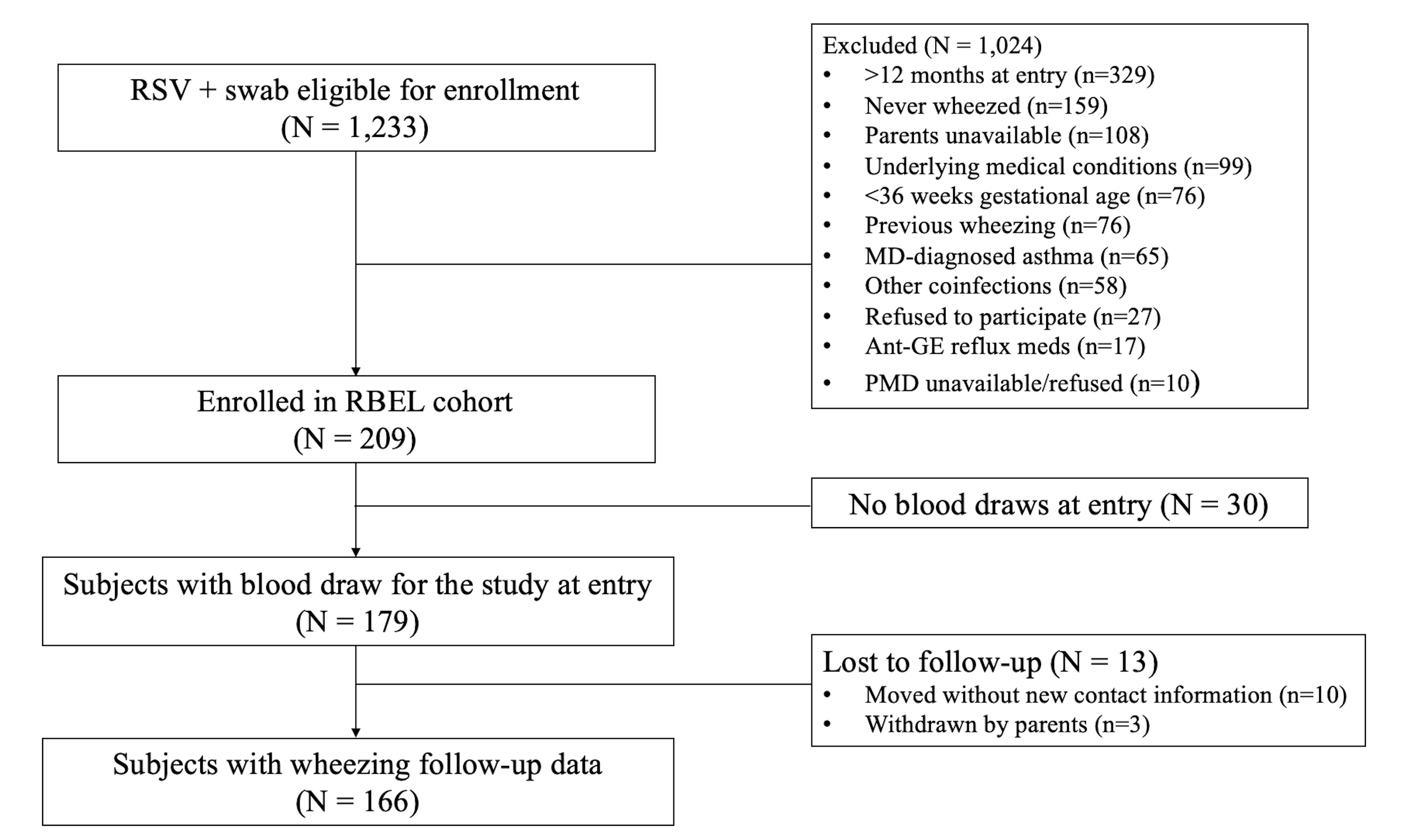

Supplement: Supplementary file 3 — Supplementary information [file IID3-8-30-s003.jpg]
