# Supplementary material for: Impaired tumor necrosis factor‐α secretion by CD4 T cells during respiratory syncytial virus bronchiolitis associated with recurrent wheeze
Source: Immun Inflamm Dis. 2020 Jan 4;8(1):30–9. doi: 10.1002/iid3.281 (PMC7016853; doi:10.1002/iid3.281)
Supplement: Supplementary file 4 — Supplementary information [file IID3-8-30-s004.docx]

**Supplemental Figure 1** Enrollment in RBEL study and subjects with blood draw for the studies and wheezing follow-up data.

* RBEL- RSV Bronchiolitis in Early Life; RSV- Respiratory Syncytial Virus; GE- Gastroesophageal reflux; PMD- Primary Medical Doctor; Tregs- Regulatory T cells
